# My Long Road Back to Life

**DOI:** 10.3201/eid3208.260904

**Published:** 2026-08

**Authors:** Christa Möbius-Friedmann, Philipp Schulz

**Affiliations:** Berlin, Germany (C. Möbius-Friedmann); Joseph-Kliniken–St. Joseph Krankenhaus Berlin–Tempelhof, Berlin (P. Schulz); Charité–Universitätsmedizin Berlin, Berlin (P. Schulz)

**Keywords:** Streptococcaceae, lactococcus, endocarditis, spondylitis, chemical and drug induced liver injury, zoonoses, bacterial zoonoses, case reports

## Abstract

A patient recounts her experience of surviving zoonotic endocarditis caused by the rare bacterial pathogen *Lactococcus garvieae*. She reflects on the potentially life-changing medical decisions she faced and the physical and emotional challenges of recovery. Her story highlights resilience, adaptation, and the gradual journey back to everyday normality.

We had just returned from a wonderful Baltic Sea holiday when blizzard-like pain from my hips to my heels ejected me from normal everyday life into hell. Getting out of my bed to the bathroom—no way. I did not presume at that moment, the lumbago or sciatica that hit me in September 2024 was only the preface to a longer, painful, even life-threatening story to come.

To put it in a nutshell: I was diagnosed with a virulent microbe infection at the age of 85. Responsible for an epizootic disease, highly aggressive, feared by farmers of trout and other fish, the bacteria at fault is called *Lactococcus garvieae.* In human beings, however, it’s only been globally registered ≈30 times. It was detected precisely in my new biologic heart valve, top functional since 2021, plus in another center of inflammation in my spine, which breathtaking pains had indicated weeks before. 

When doctors at St. Joseph Hospital [Berlin, Germany] cautiously tried to make the situation clear to me, I was aghast. Not to say shocked. My head started spinning: a third high-risk heart operation? Versus the Sword of Damocles and otherwise risk suffering a stroke due to the vegetation hanging from my infected mitral valve? And how would the spondylodiscitis develop? Wheelchair-bound in future? I don’t remember what was more frightening: the loss of weight down to 41 kg or the loss of hair. Unforgettable yet, the nurse trainee who came to me after her shift to wash my hair. Anyway, there were no guidelines yet against *Lactococcus garvieae* in human beings. So, what to do?

In spite of the SJK [St. Joseph Krankenhaus] Infectiology staff, experienced doctors, low-key nurses, as professional, as friendly and open-minded, all this laid me down. Until the day when Pablo, a young ambitious nurse from Argentina with whom I had chatted in Spanish several times before, approached me: “Cristina, ¿por que estás tan triste.” He asked me why I was so sad, and added, “All you need to do is answer this question to yourself: do you want to live or die?” It took me some nights to find my stance, confirmed by the hospital’s chaplain from the adjacent Chapel St. Joseph, who also helped me a lot while hospitalized. And above all: doctors had fulfilled my urgent wish—to cooperate by telemedicine with a surgeon of international experience including US clinics. He had operated on me before in a high-ranking modern heart center in Cottbus, south of Berlin. Together, we chose antibacterial treatment under close monitoring, with no medical guidelines existing yet. For me, it was a sensational experience in uncertain times.

And indeed, it worked. Fortunately, my blood tests, as well as MRI/CT scans, slowly showed better results, even though merciless lumbago pain continued, in spite of opium and other painkillers. Not amusing. My husband (COPD with a third of his lungs nonfunctional), however, in spite of two hours of car drive through half the city of Berlin, used to come to my bedside every day with a broad smile, bringing a bucket of fresh fruit and flowers. Love can soothe your heart.

After 6 weeks I could finally leave the hospital. The first day at home I was called back. My liver wasn’t okay: toxic hepatitis. Another 6 weeks of outpatient control followed. Meanwhile, I’m back home as an invalid with painkillers. The spondylodiscitis hasn’t vanished without leaving traces either. Latent pain and cramps remain, from my hips to my heels, due to damaged lumbar vertebrae and compressed nerves, with abdominal consequences. And nobody knows: will the germ come back? Before the *Lactococcus garvieae* infection, I was keen on being very active, biking and walking. Unfortunately, that’s over. I am sure other elderly people show similar symptoms without having had a life-threatening infection. That doesn’t make it any better, especially if you had still been doing pretty well before.

Looking back, life is no longer as easy as it was before the infection. However, it could have been worse. I’m lucky to be living in the green outskirts of Berlin, and very thankful because things have worked well. I spend more and more time without a rollator or any other walking aids. Also, due to 3 weeks of rehab and my daily exercises, plus nursing care, I have learned not to feel [like I’ve been] grounded for missing curfew. I have found my footing again. A gift of destiny?

Last but not least, we should mention the person who opened the door to my lifesaving rescue journey: the local orthopedic surgeon. He ordered an urgent new MRI, and after reviewing it, arranged my immediate hospitalization at SJK, one of the best clinics for infectiology in Berlin. To sum up, we—my husband, 90, and I—are deeply grateful to all the highly committed staff members of the St. Joseph Hospital Berlin who managed to rescue my life when hanging by a thread. 

Back at home I like hanging out again in our happy places, in the garden whenever possible, doing all we can to keep up mobilization. We prefer healthy food, a balanced lifestyle. Looking back, we remember my first weeks of being infected, when I was looking for comfort, a tiny spark of hope. Nothing more shocking than a presumed “helper,” medical staff, family or friend, shrugging shoulders and saying, “Sorry, can’t help you either.” Fortunately, this never, ever happened to me at the St. Joseph Hospital. So, I learned to channel my fear into something like a challenge, the challenge to survive. When waking up with a nightmare I opened a book, *The Light We Carry*, by Michelle Obama, written for “Overcoming in Uncertain Times.” To quote: “You can’t make decisions on fear and the possibility of what might happen…” And: “When we are able to recognize our own light, we become empowered to use it.” No less helpful were my favorite Latin authors like Isabel Allende or Mario Vargas Llosa. Finally, as an organ music lover, I found something turning out as a special blessing—the half-hour concerts at the adjacent St. Joseph Chapel.

Summing up, if care is true for each and every patient, it’s all the more for the weak, the most vulnerable, having diseases with no guidelines, no experience, no appropriate remedy. In the wake of my infection, we wish that doctors and laboratory technicians anywhere could be alert to new zoonoses and emerging diseases in general, especially in immunocompromised elderly or younger people. And we highly appreciate scientific research and development in this area.

